# Autologous Stem Cells Transplants in the Treatment of Temporomandibular Joints Disorders: A Systematic Review and Meta-Analysis of Clinical Trials

**DOI:** 10.3390/cells11172709

**Published:** 2022-08-30

**Authors:** Maciej Chęciński, Kamila Chęcińska, Natalia Turosz, Monika Kamińska, Zuzanna Nowak, Maciej Sikora, Dariusz Chlubek

**Affiliations:** 1 Department of Oral Surgery, Preventive Medicine Center, Komorowskiego 12, 30-106 Kraków, Poland; 2 Department of Glass Technology and Amorphous Coatings, Faculty of Materials Science and Ceramics, AGH University of Science and Technology, Mickiewicza 30, 30-059 Kraków, Poland; 3 Ortomania, Bartosza Głowackiego 6/1, 30-085 Kraków, Poland; 4 Collegium Medicum, Jan Kochanowski University, Aleja IX Wieków Kielc 19A, 25-317 Kielce, Poland; 5 Department of Temporomandibular Disorders, Medical University of Silesia in Katowice, Traugutta sq.2, 41-800 Zabrze, Poland; 6 Department of Maxillofacial Surgery, Hospital of the Ministry of Interior, Wojska Polskiego 51, 25-375 Kielce, Poland; 7 Department of Biochemistry and Medical Chemistry, Pomeranian Medical University, Powstańców Wielkopolskich 72, 70-111 Szczecin, Poland

**Keywords:** temporomandibular joint, temporomandibular disorders, intra articular injection, mesenchymal stem cells, stem cell transplantation

## Abstract

This systematic review aims to analyze the outcomes of the treatment of temporomandibular joint (TMJ) articular pain (AP) and restricted maximum mouth opening (MMO) with intra-articular administration of mesenchymal stem cells (MSCs). The inclusion criteria allowed primary studies involving AP and/or MMO pre-treatment and post-intervention values. Medical databases that were covered by ACM Digital, BASE, EBSCOhost, Google Scholar, PubMed, Scopus, and Web of Science engines were searched. The risk of bias was assessed with RoB 2 and ROBINS-I tools. The results were tabulated, plotted, and analyzed for regression. A total of 5 studies involving 51 patients/69 TMJs were identified, and 4 studies on 50 patients/67 TMJs were synthesized. Interventions were each time effective in decreasing AP and increasing MMO in a 6-month follow-up period by an average of about 85% and over 40%, respectively. Regression analysis showed a good fit of the logarithmic model for AP relief (5.8 − 0.8 ln x; R^2^ = 0.90) and MMO increase (33.5 + 2.4 ln x; R^2^ = 0.89). The results for AP and MMO were based on 3 studies in 39 patients and 4 studies in 50 patients, respectively, all at high risk of bias. The intra-articular administration of MSCs to TMJs, based on weak evidence, may be highly effective in reducing AP and improving MMO. This study received no funding.

## 1. Introduction

### 1.1. Rationale

**Figure 1:**
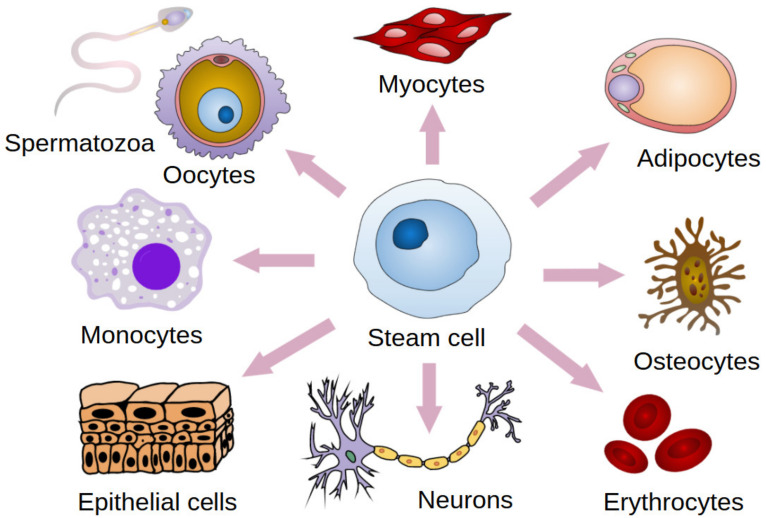
Stem cell differentiation. Modified. Haileyfournier, CC BY-SA 4.0.

Mesenchymal stem cells (MSCs) are stromal cells with a self-renewal potential [1,2,3,4]. These cells have the potential for multi-directional differentiation (Figure 1) [1,2,3]. Due to their properties such as regulation of immune responses, proangiogenic, anti-inflammatory, and regenerative effects, MSCs can exert therapeutic effects on diverse diseases [5,6,7]. They produce many immunomodulatory molecules (e.g., prostaglandin E2 and interleukin 6) [8,9,10]. Moreover they have an effect on immune system cells, inter alia, by the inhibition of monocyte maturation, proliferation, and activation of B and T lymphocytes [8,9,10]. Therefore, MSCs are used in the treatment of various diseases, such as cardiovascular diseases, spinal cord injuries, bone and cartilage repair, and autoimmune diseases [11,12,13,14]. MSCs-supported bone regeneration takes place thanks to the secretion of mediators supporting angiogenesis and osteogenesis [15]. This mechanism is used in the treatment of spinal injuries and surgery [15]. In the treatment of diabetes, the immunomodulatory effect of MSCs and their ability to improve the functioning of insulin-secreting beta cells is used [16]. Their therapeutic effect in the treatment of cardiovascular diseases is due to their ability to differentiate into vascular cells and cardiomyocytes [13]. This mechanism is behind the regeneration of the heart muscle after a myocardial infarction [13]. The use of MSCs in the treatment of neurological diseases, such as ischemic stroke or spinal cord injury, is based on the ability of these cells to stimulate myelin repair and neuron regeneration [17]. The immunomodulatory and anti-inflammatory properties of these cells suggest usefulness in the treatment of autoimmune diseases, such as Sjögren’s Syndrome [7]. In such cases it has been observed that the anti-inflammatory effect of MSCs has resulted in an improvement in the disturbed salivation process [7]. The process of cartilage regeneration can be supported thanks to the properties of MSCs, such as the modulation of inflammation, influencing the microenvironment, and the release of repair factors [18]. Due to the properties of adipose-derived stem cells, such as immunomodulatory and anti-inflammatory activity, autologous transfer is promising [19,20,21]. Targeting MSCs differentiation at the formation of cartilage tissue is currently the subject of experimental research [22,23]. Favorable conditions can stimulate the differentiation of MSCs towards chondroblasts which is promising for the treatment of cartilage degradation [22,23,24].

**Figure 2:**
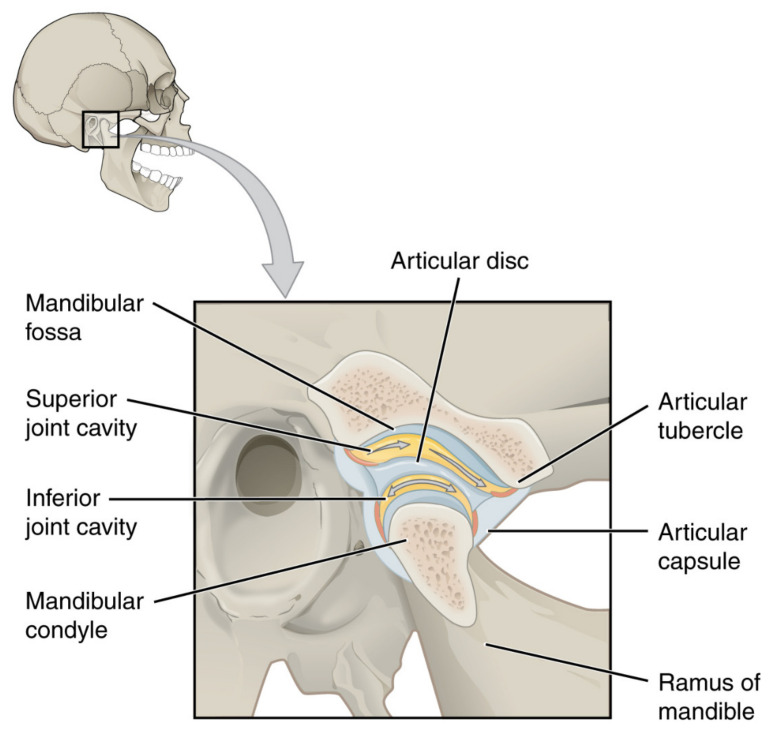
Temporomandibular joint. OpenStax College, CC BY 3.0.

**Figure 3:**
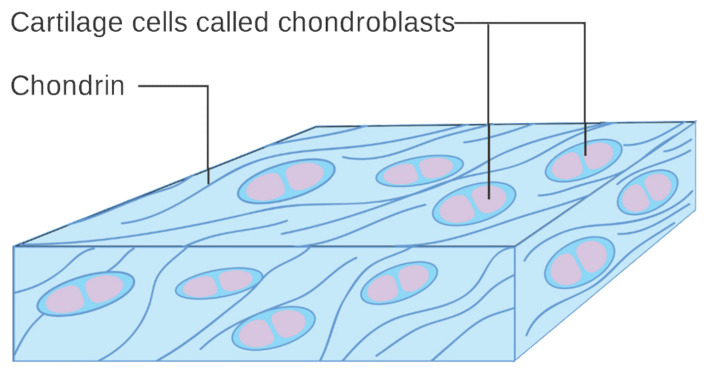
Diagram of cartilage cells called chondroblasts. Cancer Research UK, CC BY-SA 4.0.

The temporomandibular joint (TMJ) actually consists of two separate functional joints due to the existence of an articular disc (Figure 2). The joint between the temporal bone and the articular disc is the upper cavity of the TMJ. Similarly, the lower TMJ cavity has articular surfaces on the disc and head of the mandible. Originally, the articular disc is a dense fibrous tissue, which changes to fibrocartilage with age (Figure 3). Also, the above-mentioned surfaces of the bones are covered with cartilage. These structures are covered with a joint capsule and are immersed in the synovial fluid. The synovial fluid consists largely of hyaluronan, and its role is to lubricate the surfaces that are rubbing against each other. For various morphological and physiological reasons, the function of this complex system can be disturbed [25]. The characteristics of TMJs disorders (TMDs) includes inflammation and degeneration [26,27]. TMDs are caused by multiple reasons, such as malocclusion, malformations of the structure of the temporomandibular joint, degeneration of the joint surfaces and/or articular disc, and muscle and ligamentous apparatus disorders. These may result in restricted movement of the mandible and articular pain [28]. The pain itself, along with inflammatory processes, further limits the opening of the mouth [26,27,29]. 

Treatment of the progressive degeneration of TMJ is not easy, and therapeutic protocols are still under development. Open access to the TMJ is technically difficult, and even with the use of minimally invasive techniques, there is a risk of serious complications [30,31]. The gradual reduction in the invasiveness of procedures on the TMJ led to the development of arthroscopy, and as the next step, blind intra-articular injections [30,31]. Intra-articular injections into the temporomandibular joint have been used in the case of pain that is attributed to disc displacement with and without reduction, arthritis, and degenerative joint disease. [32,33]. Therefore, the effects of administration of various substances into TMJs cavities are being investigated [27,32,33,34,35,36,37]. The most frequently reported results are from the use of hyaluronan, corticosteroids, blood products, analgesics, and dextrose [35]. The supplementation of hyaluronic acid reduces the friction between the articular surfaces, which limits degenerative changes and alleviates pain [26,38]. The use of self-derived platelet-rich plasma in injections aims to regenerate damaged tissues [27,39]. Thanks to the action of growth factors, such a therapy, results in the enhancement of articular cartilage [27,39]. Intra-articular injections of various substances turned out to be effective by improving the mobility of the mandible and reducing articular pain [32,33,35]. Their use is associated with low risk of local and general complications, few of which can be considered severe [33,35,40,41,42,43,44,45,46]. Thanks to specific stem cells properties, they potentially can effectively reduce the ailments that are related to the dysfunction of the TMJs and are currently considered as another injectable [4,47,48,49].

It has been proven that MSCs are naturally present in both the cartilage and the synovial fluid of TMJs [47,50,51,52,53]. This may indicate the constant regeneration of the structures of this joint on a microscopic scale [47,50]. Just as visco-supplementation with hyaluronic acid supplements its deficiency in the synovial fluid, the administration of MSCs can supplement chondroblasts deficiencies and stimulate cartilage regeneration [4,24,26,47,54]. It is assumed that intra-articular administration of such preparations will allow the reconstruction of damaged disc cartilage and articular surfaces [4,24,47,48,49]. The displacement of the articular disc leads to a secondary inflammatory-degenerative arthropathy [55]. In such cases, injection therapies, including intra-articular administration of MSCs, are effective in relieving pain and increasing the range of motion of the mandible [55,56,57]. The reason for this effectiveness is the reduction of inflammation and the regeneration of the articular surfaces [55]. Stimulating the differentiation of MSCs towards chondroblasts is a foreign subject of research, and the results of these already published studies have shown the influence of chemical substances and physical conditions [54,58,59]. MSCs can be obtained autogenously from adipose tissue, bone marrow, and umbilical cord [24,60,61]. These are structures that are abundant in MSCs, and the selection of their source for the treatment of TMJs is largely due to practical reasons, i.e., the ease of retrieval and preparation for injection [61]. The collection of adipose tissue seems to be the best for the discussed needs due to the high content of MSCs in the preparation and the collection technique which is relatively easy and safe [61].

The first known article about MSCs transplantation into TMJs was published in 2013 [24]. In its course, it was possible to collect bone marrow MSCs, differentiate them in vitro towards chondrocytes and implant them into the joints of animals [24]. A systematic review of the use of stem cells in the treatment of TMJs was published in 2021 by Pagotto et al. [62]. These researchers qualified six animal studies and two clinical studies [62]. During the initial searches in preparation for this paper, it was found that adopting different criteria allowed for the inclusion of more human studies and thus for other systematic review objectives.

### 1.2. Objectives

This systematic review aims to identify the clinical trials and cases investigating the treatment of temporomandibular disorders by administering the stem cells into joint cavities and quantitatively evaluating the results of these reports.

## 2. Methods

This systematic review has been developed in accordance with the PRISMA methodology [63]. Checklists for the content of the report and its abstract can be found in Supplementary Documents S1 and S2, respectively [63].

### 2.1. Eligibility Criteria

The inclusion criteria allowed the clinical trials and case reports in which treatment of temporomandibular disorders was based on administering autologous stem cells into temporomandibular joint cavities. The effectiveness of the therapy was assessed in the domains of mandible mobility and changes in the intensity of joint pain. Details of the inclusion criteria according to the PICOTS methodology are presented in Table 1 [64].

### 2.2. Information Sources and Search Strategy

The searching of medical databases was carried out using four open access search engines: ACM Digital, BASE, EBSCOhost, Google Scholar, PubMed, Scopus, and Web of Science [65,66,67,68,69,70,71]. All searches were made on 22 June 2022. The following search strategy was used: temporomandibular AND (intra-articular OR injection OR injectable) AND (transplants OR stem). The queries developed for each search engine are shown in Table A1.

### 2.3. Selection Process and Data Collection Process

Reports were selected on the basis of the above-specified inclusion and exclusion criteria. First, the records that were obtained from medical databases were entered into the Rayyan tool [72]. Using the above-mentioned software, one of the authors (K.C.) performed an automatic deduplication process. Then, two authors (M.C. and K.C.) performed manual deduplication. In the next stage, the same authors screened the abstracts of all the records. The compliance assessment of both judges at the screening stage was performed using Cohen’s kappa coefficient (*k*). In the event of a discrepancy in the ratings, a given record was transferred to the full-text evaluation. The qualification on the basis of the full content of the reports was made independently by two authors (M.C. and N.T.). In the event of further discrepancies, the third judge (K.C.) had the decisive vote. Data collection was performed independently by two authors (M.C. and N.T.) without the use of automation tools.

### 2.4. Data Items, Study Risk of Bias Assessment, and Synthesis Methods

The following data characterizing the patient groups were extracted from the reports: (1) the number of injections per joint; (2) study group size; (3) the number of females; (4) the number of males; (5) patients’ age; (6) average age; (7) the number of patients that were treated unilaterally; (8) the number of patients that were treated bilaterally; (9) the number of joints treated; and (10) co-interventions. For synthesis and meta-analysis (1) maximum mouth opening (MMO) that was measured in millimeters between the incisors and (2) spontaneous pain on the Visual Analogue Scale (VAS) that was measured before the intervention and during the observation period were extracted from the reports. If the variables were assessed by other scales, the values were not converted. When converting years, months, and weeks into days for the purposes of the analyses, it was assumed that a month consists of 4-weeks. The effects were measured by the differences between the individual values that were expressed in absolute terms and as a percentage of the initial value. The risk of bias for randomized controlled trials was assessed using a revised Cochrane risk of bias tool for randomized trials (RoB 2 tool) [73]. For non-randomized studies, the risk of bias in non-randomized studies—of interventions (ROBINS-I) tool was used [74]. The risk of bias was not assessed for the case reports as they were presented separately and were not included in the synthesis or meta-analysis. Independent assessments of the risk of bias were made by two authors (M.C. and K.C.). The data were compiled in tables and then plotted on charts by two authors (M.C. and N.T.). For the assessment of statistical significance, a two-sided paired Student’s t-test was used; the test probability *p* = 0.05 was adopted. Regression analysis was attempted on the scatter plots. The Google Sheets software (Google LLC, Mountain View, CA, USA) and Microsoft Excel (Microsoft Corporation, Redmond, WA, USA) program was used for visualization and analysis.

## 3. Results

### 3.1. Study Selection

**Figure 4:**
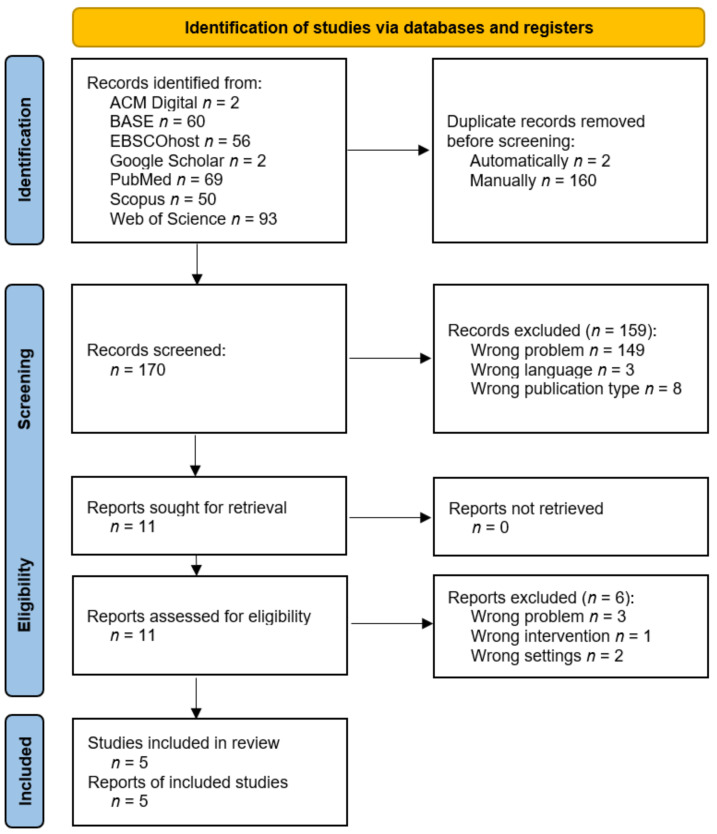
PRISMA flow diagram.

In total, 332 records were found and 2 of them were deleted in the automatic deduplication process. A manual review of duplicates led to the removal of another 160 items. The remaining 170 abstracts were screened and 11 of them were qualified for full-text evaluation (*k* = 0.83). Ultimately, five studies that were described in five reports were included for the synthesis. Details of the selection process are presented in Figure 4. The reports that were rejected at the full-text analysis stage are summarized with exclusion reasons in Table A2.

### 3.2. Study Characteristics

Reports that qualified for synthesis are listed in Table 2 together with the characteristics of the studied groups. In all studies, a single intra-articular administration of stem cells was performed, i.e., the intervention was not repeated. In total, the treatment with stem cells of 69 TMJs in 51 patients was described in the identified reports. Table A3 shows the control groups in the reports in which they were described.

The report by Carbonini et al. describes a study that was conducted in Italy concerning eight patients who were randomized into two equal groups: the study group and the control group. All patients suffered from internal derangement in the TMJ. The inclusion criteria were: (1) headache or joint pain (VAS ≥ 4), (2) joint noise and limited mouth opening, (3) patients with previously conservative treatment without symptoms resolution, (4) symptoms lasting for more than 2 years, and (5) MRI that was positive for capsular-ligamentous structures diseases. The study group patients (three males, one female) were treated with arthrocentesis and fat-derived stem cell injections (1 mL). There were seven joints that were treated in total (in three patients they treated both TMJ, whereas in one patient—only the right side). For 15-days, patients took NSAIDs, followed a bland diet, and did physiotherapy exercises. The control group consisted of four patients treated with saline injections to the upper compartments (supra of both TMJs. The patients returned for follow-up after 1-week, 1-month, 3-months, and 6-months. The progressive improvement was observed in pain and movement. The average level of pain decreased more in the study group than in the control group. The MMO increased by 5.75 mm in the study group and by 3.25 mm in the control group [55].

In the study of De Riu et al., a total of 30 patients aged 33–67 years old were enrolled. The patients that were affected by severe unilateral temporomandibular joint disorders with internal derangement, were randomly divided into two groups of 15. Patients in the control group (14 females, 1 male) underwent arthrocentesis using Ringer’s lactate solution plus intra-articular injection of hyaluronic acid. Patients in the study group (15 females) received an injection of 2 mL bone marrow nucleated cell concentrate. The patients returned for follow-up after 1-week, 1-month, 6-months, and 1-year. In both groups, the pain during motion was significantly reduced. After 1-year, the average VAS pain during motion decreased more in the study group than in the control group. The VAS pain at rest also changed from 8.2 to 1.87. The maximum interincisal opening improved after the procedure as well, by 11.8 mm in the study group, and by 4.87 mm in the control group [57].

The article by Mahmmood et al. describes the study involving 11 patients (eight females, three males) with temporomandibular disorders. There were six inclusion criteria: (1) clinical diagnosis of anterior disc displacement, (2) limitation in mouth opening, (3) periauricular pain, (4) the presence of symptoms for at least 3-months, (5) clicking, and (6) deviation during mouth opening. The procedure was performed on a single joint in three patients, and bilaterally in eight patients. Before the injection, 8 out of 11 patients presented with pain, and five patients had a problem with limitation in mouth opening. The follow-up period was every 2-weeks for the first 3-months and then monthly. After the 2-weeks, pain disappeared among all the patients and MMO improved (from 28.2 mm to 34.4 mm). There were no significant postoperative complications, apart from mild pain because of the injection and mild bruising on the donor site in one patient. 

Sembronio et al. reported 40 patients (31 females, 9 males) between 17 and 74 years old. They were randomized into the control group consisting of 20 patients who underwent arthrocentesis with intra-articular injection of hyaluronic acid (HA) and the study group including 20 patients (6 bilateral, 14 unilateral) who received an injection of microfragmented adipose tissue (2 mL) from the abdominal wall. One patient had the harvesting procedure performed in the medial thigh due to a deficiency of abdominal fat. The pain and maximum interincisal opening were evaluated at follow-up examination 10-days, 1-month, and 6-months after the procedure. After 6-months, the pain decreased more in the study group than in the control group. The MIO also improved more in the study group (by 11.7 mm) than in the control group (by 6.2 mm).

In the case report that was published by De Souza Tesch et al., a regenerative medicine approach was proposed using in vitro expanded autologous cells from the nasal septum. A 27-year-old male had a severe skeletal Class II and mandibular micrognathia, with an indication of orthognathic surgery for its correction. The CT images showed degenerative changes in both the temporomandibular joints, with intense condylar resorption, especially in the right one. Before the treatment started, the von Korff index for chronic pain severity showed a low pain intensity (<50) without disability (grade I), and the MMO was 23 mm. No complications were reported. After 2 weeks, the MMO improved to 30 mm. After 1-month, the von Korff index of chronic pain severity was zero, and the MMO had a mild decrease to 27 mm. Three months later, the patient still did not feel any pain (the von Korff scale persisted at a score of zero), and the MMO improved to 31 mm. After 6-months, the MMO improved by 1 mm, and the von Korff index did not change. After 1-year, there were no complaints of pain at rest or during motion, and the assisted MMO reached 36 mm.

### 3.3. Risk of Bias in Studies

The risk of bias was assessed for three randomized trials and one non-randomized trial. Each of the studies was characterized by a high risk of bias, which was mainly due to the fact that it was impossible to conceal patients’ membership of the study or placebo group from participants and surgeons delivering the interventions. A detailed assessment is presented in Table 3.

### 3.4. Results of Individual Studies

The results of individual studies in the domains of joint pain relief and increasing the extent of mandibular abduction are presented in Table 4 and Table 5, respectively. The percentage of the initial value of the variable is given in parentheses. The article by de Souza Tesch et al. was a single case report, and, therefore, its results could not be included in the synthesis. For this reason, they were not taken into account when calculating the mean values of MMO and are included in the last row of the respective table.

### 3.5. Results of Syntheses

#### 3.5.1. Pain

**Figure 5:**
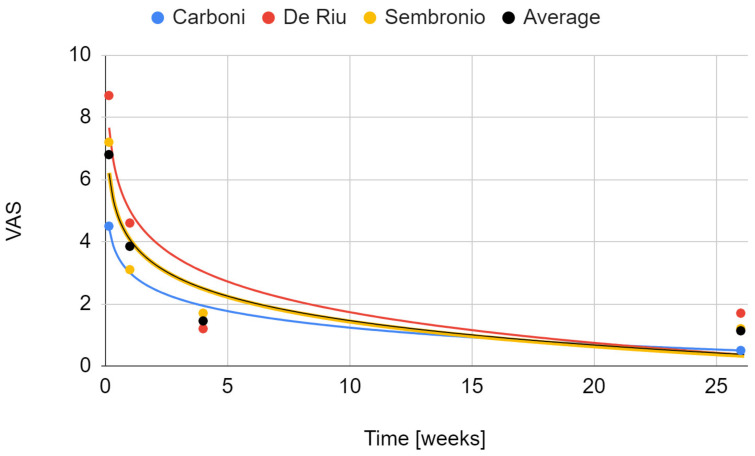
Change in the VAS joint pain intensity over time.

**Figure 6:**
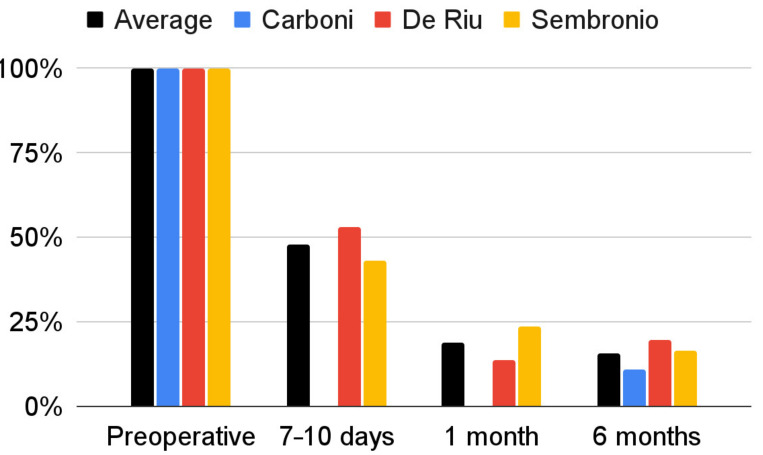
Percentage change in the VAS joint pain intensity over time.

The severity of joint pain decreased during the follow-up after the intervention. The data that were needed for this synthesis were provided by the reports by Carboni et al., De Riu et al., and Sembronio et al., whose results appear to be similar [55,57,75]. For the De Riu study, the severity of pain in motion was taken into account as Sembronio et al. used similar measurement conditions [57,75]. A greater analgesic effect was observed in the first days after the intervention [57,75]. As follow-up, pain relief was progressive, but with less and less intensity [57,75]. Regression analysis showed that both the results of individual studies by De Riu et al. and Sembronio et al., as well as the averaged values for the three reports, the logarithmic model is the closest in the 6-month observation (4.07 + −1.13 ln x; R^2^ = 0.90) [55,57,75]. These results are presented graphically in Figure 5. The percentage changes in the value of pain intensity during the 6-month follow-up are presented in Figure 6. 

#### 3.5.2. Maximum Mouth Opening

**Figure 7:**
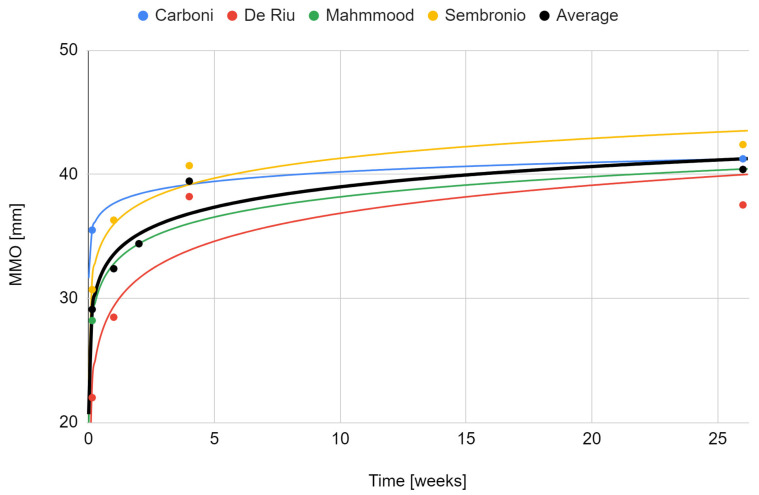
Change in the maximum mouth opening over time.

**Figure 8:**
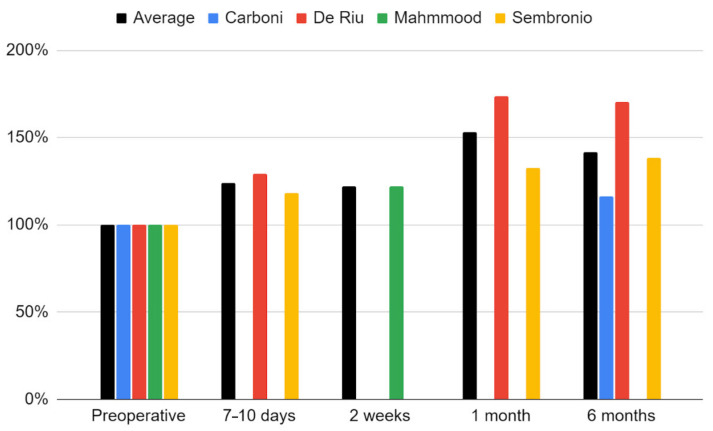
Percentage change in the maximum mouth opening over time.

The maximum mouth opening variable that was examined in all qualifying reports [55,56,57,75,76]. Due to a weaker level of evidence (single case), the study by de Souza Tesch et al. was not taken into account in this synthesis [76]. The range of mandibular mobility increased during post-intervention observation. Only in the report by De Riu et al. was there a decrease in relation to the previous values [57]. It was the study with the longest follow-up (one year), and the loss of therapeutic effect in this domain occurred in the last phase of observation [57]. The strongest effect was seen in each report immediately after the intervention [57,75]. Again, the fit of the logarithmic regression model for 6-months of observation turned out to be the closest (33.5 + 2.37 ln x; R^2^ = 0.89) (Figure 7). The percentage increase in the mouth opening range in individual studies and as the mean of the meta-analysis is presented in Figure 8.

#### 3.5.3. Comparison with Control Groups

**Figure 9:**
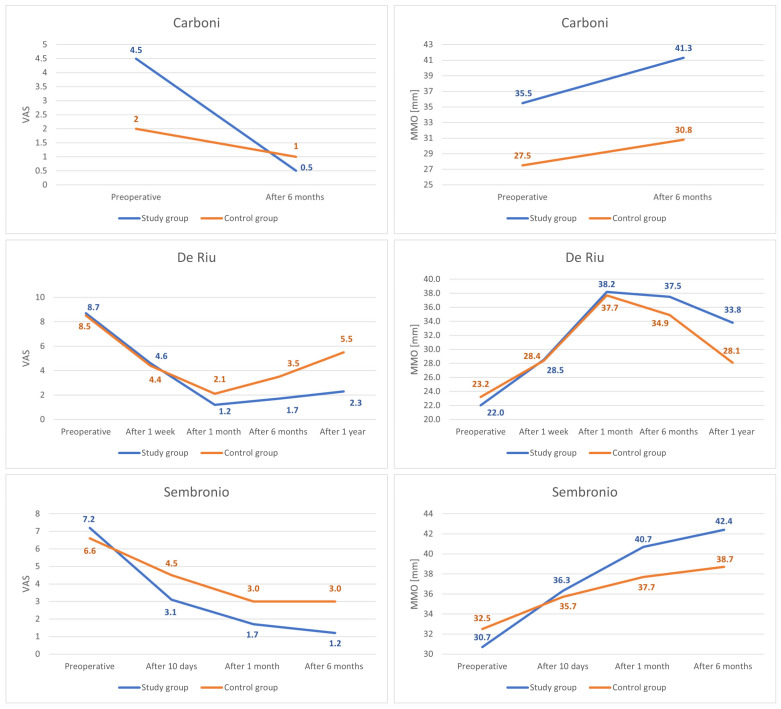
Comparison of VAS pain and MMO values for the study and control groups.

The VAS pain values in each of the controlled trials decreased more in the study group than in the control group over the course of 6-months [55,57,75]. The differences in the final and initial discrepancies between the groups in the studies by Carboni et al., De Riu et al., and Sembronio et al. were −3, −2, and −2.4, respectively, on average −2.5, considered statistically significant (*p* = 0.014). [55,57,75]. The same reports showed a stronger increase in the MMO values after 6-months in the study groups compared to the control groups [55,57,75]. The differences in the final and initial discrepancies in the studies by Carboni et al., De Riu et al., and Sembronio et al. were 2.5 mm, 3.8 mm, and 5.5 mm, respectively, on average 3.9 mm, considered statistically significant (*p* = 0.045). [55,57,75]. The detailed course of comparative observations for the above-mentioned studies is presented graphically in Figure 9 [55,57,75].

## 4. Discussion

### 4.1. Interpretation of the Results

All of the studies that were identified in the course of this systematic review showed a reduction of pain and an increase of the MMO due to the intra-articular injections of stem cells [55,56,57,75,76]. The pain level was measured with the VAS scale in three studies [55,57,75]. After 6-months, the mean decrease in pain level was VAS 5.68. The largest reduction of pain (7 points) was observed in the study by De Riu G. et al. whereas the lowest reduction of pain (4 points) was measured in the study by Carbonini et al. [55,57]. In four studies, the MMO was controlled after 6-months and the average increase of the MMO was 12.05 mm [55,56,57,75]. The largest increase in the MMO (15.53 mm) was observed in the study by De Riu G. et al. where the patients received an injection of 2 mL bone marrow nucleated cell concentrate (BMNc) [57]. This can be possibly explained by the fact that preoperatively, the maximum mandibular abduction was the smallest among all of the studies [57]. The lowest increase (6.2 mm) was observed in the study by Mahmmood et al. which is probably due to the lack of arthrocentesis [56]. In addition, the variable was measured only 2-weeks after the injection of nano-fat [56]. In the control groups, after 6-months, there was an increase of the MMO by an average of 7.06 mm, and a decrease in pain by an average of 3.22 points on the VAS scale [55,57,75]. The results that were observed in the study groups were noticeably better than in the control groups [55,57,75]. The De Riu et al. study is the only one to illustrate further observation [57]. It was reported that both joint pain and mandibular mobility deteriorated one year after the intervention as compared to the value of 6-months after the intervention [57]. Therefore, it can be assumed that the best results of therapy with MSCs injections into TMJs are achieved about 6-months after the intervention, and then a gradual deterioration occurs. It should be emphasized, however, that the results for pain and abduction were still impressive after one year, about 25% and about 150% of the baseline values, respectively.

### 4.2. Treatment Technique

As shown by the modest results of this review, the injection of autologous stem cells into the cavities of TMJs is not popularly performed. The treatment technique that is used by different researchers differs and it can be assumed that it is at the stage of development [55,56,57,75,76]. In the study by Carbonini et al., the solution that was used for intra-abdominal administration (for the subsequent receive and gain of fat-derived stem cells) was prepared using 250 mL of saline, 20 mL of lidocaine, and 0.5 mL of adrenaline, and afterwards 30mL of adipose cells were acquired. Then, a dedicated kit was used to obtain the filtrate containing 1mL of mesenchymal stem cells, which were inserted into the upper compartment of TMJ [55]. De Riu et al. describes the extraction of bone marrow nucleated cell concentrate from the iliac crest under local anesthesia. The donor site was located at least 2 cm behind the anterior superior iliac spine. About 30 mL of medullary blood was withdrawn with a sharp trocar and then transferred to a concentration tube, which was centrifuged at 3200 revolutions per minute for 15 min. The cell-poor plasma was eliminated, while the BMNc concentrate was drawn into a sterile syringe and injected inside the upper joint space [57]. The source of the stem cells in the report of Mahmmood et al. was nano-fat. The following was used to prepare the tumescent solution: 1000 mL of ringer lactate solution, 12.5 mL of 2% lidocaine, and 1 mL ampule containing 1g of epinephrine were mixed and applied to the lower abdomen (donor site). A volume of the solution was administered that was equal to three times the volume of fat to be withdrawn. The 3 mm incision was made on the lower lateral right or left abdominal region, and then the cannula was inserted. When the harvesting was completed, the cannula was removed, the fat was separated from other aspirated fluids, and washed with saline. To obtain nano-fat emulsion, the pure fat was transferred to a 5mL hypodermic syringe which was connected to another 5 mL hypodermic syringe and pushed 50 times between them. A total of 2 mL of the obtained material was injected into the superior joint space. After the procedure, the patients took an antibiotic (amoxicillin with clavulanate 625 mg, metronidazole 200 mg) and nonsteroidal anti-inflammatory drugs (for 7-days) and were instructed to follow a semi-soft diet for 2-weeks [56]. In the study of Sembronio et al., the amount of tumescent solution that was administered into the abdominal cavity was 120-150 mL and composition: 1000 mL of saline, 100 mL of 1% lidocaine, 1 mL of epinephrine, and 10 mL of 8.4% sodium bicarbonate. A few minutes after injecting the solution, lipoaspiration was conducted. A dedicated system was used to perform microfragmentation of adipose tissue. The fatty aspirate was pushed through the inlet filter. As the device was shaken, an emulsion of oil, blood, and saline was achieved, the latter being washed away. An additional filter is used, and subsequently, the final product is collected into a syringe that is connected to the upper opening of the device to eliminate the excess of liquid fraction [75]. In the case report of de Souza Tesch for the isolation and preparation of the nasal septum-derived chondrocytes, a nasal cartilage biopsy was performed under general anesthesia. The cartilage was minced into small fragments and digested with 0.1% collagenase solution. The obtained cells were cultured for 21-days and were cryopreserved. One week prior to injection, the cells were thawed and cultivated. Two days prior to injection, 20% fetal bovine serum (FBS) was replaced by autologous serum that was derived from the patient’s blood. The cells were suspended in phosphate-buffered saline (PBS) and placed in two cryogenic tubes. The patient received an injection (1 mL containing 107 cells) in each TMJ [76].

### 4.3. Effectiveness

Publications regarding intra-articular injections in the treatment of temporomandibular joint disorders have addressed arthrocentesis and the administration of hyaluronic acid, corticosteroids, blood products, analgesics, and hypertonic dextrose [35]. The effectiveness of these therapeutic methods is the subject of current research [32,33,35,77]. Derwich et al. compared the mechanism of action of hyaluronic acid, corticosteroids, and platelet-rich plasma that was administered in the form of injections in the treatment of temporomandibular joint dysfunction. Hyaluronic acid has a protective effect on cartilage and inhibits its degradation as well as it has anti-inflammatory properties, increases hydration, stimulates the synthesis of proteoglycans, and by affecting nerve conduction contributes to the reduction of pain, which may be in some aspects similar to the action of stem cells [78]. Sit et al. discusses the aspect of hypertonic dextrose prolotherapy. Its presumed effect is inducing an inflammatory reaction, and thus stimulating tissue proliferation and acceleration of healing. The similarity in the action of hypertonic dextrose prolotherapy and fat-derived stem cells is the presumed mechanism of action by influencing cells of the immune system and stimulating tissue regeneration, both of which reduced pain in most of the treated patients [77]. The legitimacy of using preparations containing stem cells was compared to control groups in three studies [55,57,75]. A study by Sembronio et al. showed a statistically significant superiority of stem cell therapy over hyaluronan in the context of pain relief and increasing the mobility of the mandible [75]. In both groups, the administration of preparations was preceded by arthrocentesis [75]. The same comparison confirmed the better analgesic effect of stem cell treatment in the report by De Riu et al. [57]. Only in the study by Mahmmood et al., arthrocentesis was not performed as part of the intervention [56]. The results of this decision cannot be directly compared with other reports due to the different observation times [55,56,57,75]. Nevertheless, based on the changes in MMO in this and other studies, it can be assumed that the use of arthrocentesis probably increases the effectiveness of therapy in this domain [55,56,57,75].

In this systematic review, we identified only three reports comparing the efficacy of intra-articular administration of MSCs to other injectable therapies [55,57,75]. In two studies, the control group was administered hyaluronic acid, and in the third, only arthrocentesis was performed [55,57,75]. De Riu et al., who compared MSCs to hyaluronan, noted the greatest improvement in VAS pain and MMO after 1-month of treatment [57]. After one year, there was a decrease in VAS pain of 74% in the study group and 35% in the control group that were treated with hyaluronic acid injection [57]. The MMO increased by 11.8 mm (35%) in the study group and by 4.9 mm (17%) in the control group [57]. Sembronio et al., after 6-months of hyaluronan-controlled trial, reported a greater decrease in VAS pain in the study group (by 83%) than in the control group (by 55%) [75]. MMO increased by 11,7 mm (28%) in the study group and in the control group by 6,2 mm (16%) [75]. In the report by Carbonini et al., after 6-months of treatment, there was a greater decrease in VAS pain in the MSCs group (by 89%) than in the arthrocentesis group (by 50%) [55]. MMO increased by 5.8 mm (16%) in the study group and by 3.3 mm (12%) in the control group [55].

Carboni et al. added that the administration of fat-derived stem cells also improved the morphology of the articular structures that were imaged by magnetic resonance [55]. Computed tomography imaging studies have produced consistent results in an animal model, demonstrating an improvement in TMJ cartilage morphology of the mandibular head due to the administration of MSCs [79,80]. For comparison, in studies of the administration of MSCs to the knee joints, morphological changes in the cartilage were observed on magnetic resonance imaging (MRI) [81,82]. In the study of Vega, patients in the test group who suffered from knee osteoarthritis, received an intra-articular injection of allogeneic bone marrow MSCs [81]. The cartilage improvement was confirmed by the MRI quantitative T2 mapping—the quality of cartilage improved, whereas the number of poor cartilage areas decreased [81]. The report by Park showed the positive influence on cartilage regeneration in patients who suffered from osteoarthritis that were treated with MSCs that were obtained from umbilical cord blood [82]. The MRI performed 3-years after the procedure was done, showed persistent well-healed cartilage [82].

### 4.4. Complications

Possible unfavorable directions for stem cell differentiation, including proinflammatory phenotype-producing pro-inflammatory cytokines, are being cautioned about [83,84,85,86]. The issue requires further investigation but does not appear to be a deterrent to clinical trials. Human studies did not reveal many macroscopically noticeable complications, and analysis at the tissue level was not performed. In the study of Sembronio, a mild hematoma in the abdominal wall in three patients that did not persist at a later follow-up was reported [75]. Mahmmood et al. reported pain in the recipient site, and in one patient moderate bruising of the donor site [56]. Obtaining stem cells that are derived from nano-fat from the abdomen is generally preferred over bone marrow aspiration due to the fact it has a lower risk of side effects, it is easier to perform and provides a richer source of stem cells [87]. De Riu et al. stated that no major complications have been noted, but give no details [57]. De Souza Tesch describes no complications in the reported case [76]. However, it should be remembered that any transplantation of adipose tissue carries the risk of mistaken intravascular fat injection, which may lead to embolism and further consequences [88]. Injections of other substances into the cavities of the temporomandibular joints resulted in numerous mild and semi-severe postoperative complications. The mild ones included swelling, pain, pressure in the ears, and local rashes [42,43,44,45,46]. The more serious ones were generalized rashes, fever, open bite, mandibular hypomobility, malocclusion, skin hypopigmentation or atrophy, and hypoesthesia [42,43,44,45,46]. Another failure of intra-articular injections may be overtreatment due to the misdiagnosis of joint and muscle pain [26,89,90]. The latter ought to be treated with transdermal medications, intramuscular injections of drugs, or dry needling [89,90,91,92].

### 4.5. Differential Diagnosis

MSCs transplantation cannot be treated as a remedy for all ailments that are related to the limited mobility of the mandible. Differential diagnosis takes into account (1) injuries, (2) missing teeth, (3) disturbed occlusive conditions, and (4) excessive muscle tension [31,89,91,93,94,95,96]. Fractures of the mandibular head and condylar neck can be difficult to diagnose, and if confirmed, they pose a therapeutic challenge [30,31,94,97]. For a clinician considering the treatment of joint pain and restricted mouth opening with MSCs injections, the key is to exclude not only acute trauma, but also the post-trauma condition, as the cause of the ailment may be dislocation of healed bone fragments, developing TMJ ankylosis, and chronic irritation with protruding osteosynthetic material [30,31,94,97]. Missing teeth and occlusal disorders lead to abnormal loads in TMJs and can cause functional disorders that, in the long run, turn into morphological defects [98]. TMJ is self-healing to some extent and causal treatment may be sufficient. In such situations, MSC transplants could prove to be overtreatment. As an emergency, physiotherapy, splint therapy, pharmacotherapy, arthrocentesis, and intra-articular hyaluronan should be considered first, as these methods do not require a donor site [91,95,98]. Good results in pain relief in patients that were diagnosed with temporomandibular disorders have also been shown for infrared light photobiomodulation [99,100,101,102,103]. Increased muscle tension can result in muscle pain that is difficult to differentiate from joint pain the more that the limitation of opening the mouth also coexists [26,91,96]. The cause of masticatory muscle pain may not only be purely somatic, but also to some extent psychogenic [26]. There are a number of treatments for the masticatory muscles, including dry needling botulin toxin injections, and the administration of relaxation medications [89,90,92,96,104,105].

### 4.6. Further Research

Embree et al. discussed the use of resident fibrous cartilage stem cells in cartilage regeneration, which could potentially be used in the treatment of temporomandibular joints [50]. These authors suggested that the place of origin of the collected cells, the type of joint into which they will be administered, as well as the intra-articular co-application of other substances may affect the properties and therapeutic effect of stem cells [50]. The regenerative potential for the structures of the temporomandibular joint was confirmed in in vitro studies [106]. Intra-articular stem cell injections have repeatedly shown good results in animal studies [107,108,109,110,111]. It has been shown in an animal model that not only direct injection of the preparation containing stem cells, but also the use of carriers gives good regenerative effects [111]. The possibility of using MSCs in the treatment of pain and functional limitations of the knee joint has been confirmed in humans [81,82,112]. It is suspected that such an intervention may lead to the regeneration of the knee cartilage [81,82,112]. Also of clinical importance is also the possibility of collecting stem cells from sources other than adipose tissue and bone marrow [113,114,115]. Peripheral blood, dental pulp, and periodontal ligaments are also considered as their source [113,114,115]. However, the use of MSCs poor donor sites requires their multiplication in in vitro culture [113,114,115]. Stem cell preparations are just one of the many injectables that are potentially useful in the treatment of TMJs [26,32,33,35]. However apart from preceding the administration of MSCs with arthrocentesis, there are currently no known combination therapies that have been tested in humans. Of the blood-derived substances that are also a kind of autologous transplant platelet-rich plasma, injectable platelet-rich fibrin and plasma that is rich in growth factors that is injected with and without rinsing the joint have provided good results [32,35]. Other developing treatment strategies for temporomandibular disorders include intra-articular and intramuscular drugs injections and the eligibility for each depends on the diagnosis and clinical symptoms [35,89,90,91,92,93,98,104].

### 4.7. Limitations

One study was not included in the synthesis as it was a case report and thus did not present a sufficient level of evidence [76]. All of the remaining studies were at high risk of bias [55,56,57,75]. Therefore, the results for articular pain and MMO were based on 3 studies in 39 patients and 4 studies in 50 patients, respectively [55,56,57,75]. The wide variety of observation times for the reported variables represents an additional limitation of the evidence [55,56,57,75].

## 5. Conclusions

The intra-articular administration of mesenchymal stem cells to temporomandibular joints, based on weak evidence, may be highly effective in reducing articular pain and improving maximum mouth opening in temporomandibular disorders.

## Figures and Tables

**Table 1:** Eligibility criteria.

	Inclusion Criteria	Exclusion Criteria
Problem	Human patients with temporomandibular joint disorders	Orthognathic surgery, mandibular condyle fracture, and ankylosis cases
Intervention	Intra-articular injections with stem cells	Non-autologous transplants, injections of other substances
Comparison	Any or none	-
Outcomes	Joint pain and mandibular mobility assessments	No initial values of the variables provided
Timeframe	No limits	
Settings	Primary studies	Non-English reports

**Table 2:** Characteristics of the studied groups. F-females; M-males; N/S-not specified.

First Author	Study Group Size (F/M)	Patients’ Age (Average)	Patients Treated Unilaterally/Bilaterally	Number of Joints Treated	Co-Intervention
Carboni [55]	4 (1/3)	N/S (N/S)	1/3	7	Arthrocentesis
De Riu [57]	15 (15/0)	35–67 (48.2)	15/0	15	Arthrocentesis
Mahmmood [56]	11 (8/3)	18–34 (24.1)	3/8	19	None
Sembronio [75]	20 (N/S)	17–74 (43.3)	14/6	26	Arthrocentesis
de Souza Tesch [76]	1 (0/1)	27 (27)	0/1	2	Arthrocentesis

**Table 3:** Risk of bias in studies: (1) arising from the randomization process, (2) due to deviations from the intended interventions, (3) due to missing outcome data, (4) in measurement of the outcome, (5) in the selection of the reported result, (6) due to confounding, factors, (7) in selection of participants into the study, and (8) in the classification of interventions. RT—randomized trial; NRSoI—non-randomized study—of interventions; S/C—some concerns; N/A—not applicable.

First Author	Study Type	(1)	(2)	(3)	(4)	(5)	(6)	(7)	(8)	Overall Risk of Bias
Carboni [55]	RT	Low	High	Low	S/C	Low	N/A	N/A	N/A	High
De Riu [57]	RT	Low	High	Low	Low	Low	N/A	N/A	N/A	High
Mahmmood [56]	NRSoI	N/A	Low	High	Low	Low	Low	Low	Low	High
Sembronio [75]	RT	Low	High	Low	S/C	Low	N/A	N/A	N/A	High

**Table 4:** Results of the individual studies in the domain of VAS pain relief.

First Author	Group	Preoperative	After 7–10-Days	After 1-Month	After 6-Months	After 1-Year
Carboni [55]	Study group	4.5 (100%)			0.5 (11%)	
	Control group	2 (100%)			1 (50%)	
De Riu [57]	Study group	8.7 (100%)	4.6 (53%)	1.2 (14%)	1.7 (20%)	2.3 (26%)
	Control group	8.5 (100%)	4.4 (52%)	2.1 (25%)	3.5 (41%)	5.5 (65%)
Sembronio [75]	Study group	7.2 (100%)	3.1 (43%)	1.7 (24%)	1.2 (17%)	
	Control group	6.6 (100%)	4.5 (68%)	3.0 (45%)	3.0 (45%)	
Average	Study group	6.8 (100%)	3.9 (48%)	1.5 (19%)	1.1 (16%)	2.3 (26%)
	Control group	5.7 (100%)	4.5 (60%)	2.6 (35%)	2.5 (45%)	5.5 (65%)
Standard deviation	Study group	2.1	1.1	0.4	0.6	
	Control group	3.3	0.1	0.6	1.3	

**Table 5:** Results of the individual studies in the domain of maximum mouth opening [mm].

First Author	Group	Preoperative	After 7–10-Days	After 14-Days	After 1-Month	After 6-Months	After 1-Year
Carboni [55]	Study group	35.5 (100%)				41.3 (116%)	
	Control group	27.5 (100%)				30.8 (112%)	
De Riu [57]	Study group	22.0 (100%)	28.5 (129%)		38.2 (174%)	37.5 (171%)	33.8 (154%)
	Control group	23.2 (100%)	28.4 (122%)		37.7 (163%)	34.9 (150%)	28.1 (121%)
Mahmmood [56]	Study group	28.2 (100%)		34.4 (122%)			
Sembronio [75]	Study group	30.7 (100%)	36.3 (118%)		40.7 (133%)	42.4 (138%)	
	Control group	32.5 (100%)	35.7 (110%)		37.7 (116%)	38.7 (119%)	
Average of the above	Study group	29.1 (100%)	32.4 (124%)	34.4 (122%)	39.5 (153%)	40.4 (142%)	33.8 (154%)
	Control group	27.7 (100%)	32.1 (116%)		37.7 (140%)	34.8 (127%)	28.1 (121%)
Standard deviation of the above	Study group	5.6	5.5		1.8	2.5	
	Control group	4.7	5.2		0	4.0	
de Souza Tesch [76]	Study group	23.0 (100%)		30.0 (130%)	27.0 (117%)	32.0 (139%)	36 (157%)

## Data Availability

The protocol of the systematic review was submitted to PROSPERO under the number: 342832. All data that are presented in this study are available in this article and its Supplementary Materials.

## Supplementary Materials

The following supporting information can be downloaded at: https://www.mdpi.com/article/10.3390/cells11172709/s1, Document S1: PRISMA 2020 Checklist; Document S2: PRISMA 2020 for Abstracts Checklist.

## Appendix A

**Table A1:** Search queries.

Search Engine	Search Query
ACM Digital	[All: temporomandibular] AND [[All: intra-articular] OR [All: injection] OR [All: injectable]] AND [[All: transplants] OR [All: stem]] Searched the ACM Guide to Computing Literature
BASE	temporomandibular AND (intra-articular injection injectable) AND (transplants stem)
EBSCOhost	temporomandibular AND (intra-articular OR injection OR injectable) AND (transplants OR stem)
Google Scholar	allintitle: temporomandibular AND (intra-articular OR injection OR injectable) AND (transplants OR stem)
PubMed	temporomandibular AND (intra-articular OR injection OR injectable) AND (transplants OR stem)
Scopus	TITLE-ABS-KEY (temporomandibular AND (intra-articular OR injection OR injectable) AND (transplants OR stem))
Web of Science	temporomandibular AND (intra-articular OR injection OR injectable) AND (transplants OR stem) (All Fields)

**Table A2:** Reports that were rejected at the eligibility stage.

First Author	Report	Reason for Exclusion
Embree [50]	Exploiting endogenous fibrocartilage stem cells to regenerate cartilage and repair joint injury	No human in vivo studies
Guarda-Nardini [116]	Human Amniotic Membrane Positioning in the Surgical Treatment of Temporomandibular Joint Degenerative Disorder	Wrong intervention
Serakinci [4]	Modeling Mesenchymal Stem Cells in TMJ Rheumatoid Arthritis and Osteoarthritis Therapy	Review article
Ward [117]	Umbilical Cord Stem Cell Lysate: A New Biologic Injection for Treatment of Temporomandibular Joint Inflammation	Animal study
Yang [24]	Bone marrow mesenchymal stem cell transplantation for the treatment of temporomandibular joint osteoarthrosis	Animal study
Zhu [118]	NEL-like molecule-1-modified bone marrow mesenchymal stem cells/poly lactic-co-glycolic acid composite improves repair of large osteochondral defects in mandibular condyle	Wrong problem

**Table A3:** Characteristics of the control groups. F—females; M—males; N/S—not specified; HA—hyaluronic acid administration.

First Author	Control Group Size (F/M)	Patients’ Age (Average)	Patients Treated Unilaterally/Bilaterally	Number of Joints Treated	Interventions
Carboni [55]	4 (2/2)	28–62 (44.0)	0/4	8	Saline injection
De Riu [57]	15 (14/1)	33–61 (44.5)	15/0	15	Arthrocentesis + HA
Sembronio [75]	20 (N/S)	17–74 (50.7)	15/5	25	Arthrocentesis + HA
